# Supplementary material for: Patterns of Intron Gain and Loss in Fungi
Source: PLoS Biol. 2004 Nov 30;2(12):e422. doi: 10.1371/journal.pbio.0020422 (PMC532390; doi:10.1371/journal.pbio.0020422)
Supplement: Table S1 — Also available at http://genes.mit.edu/NielsenEtAl/. (4.3 MB ZIP). [file pbio.0020422.st001.zip › NielsenEtAl/html/669.html]

AN1396.1.NCU05454.1.MG03147.1.FG03249.1


```
 CLUSTAL W (1.82) Multiple Sequence Alignments - Introns Inserted


Sequence 1: NCU05454.1	736 aa
Sequence 2: MG03147.1	685 aa
Sequence 3: AN1396.1	708 aa
Sequence 4: FG03249.1	686 aa
Alignment Length: 749 aa
Number Identitical Residues: 290 aa
Alignment Score (without introns) 15883


MG03147.1 	-MSRFARGSILRRLAAPTAITALGGSLVLYAYRPRNIPGFEDPAVPPPTYGADGTFKLPR
NCU05454.1	-MSRLLR---AKKLVAPALVAGVAGGSLYYMYKPRNIPGYEGPVVPPPIFGADGTFKLPR
FG03249.1 	-MQQRLRSMKRPLLLAAAATTTLAVGGGYYATRNR--THVVDKALVPLKRDQTGRIVPPT
AN1396.1  	MASRNTRRILRPLLYGTAAVTTVGAGALYFSIRPRNIPGLEAPAVPPPGY-HEGKLVPPS
          	  .:  *      * ..:  : :. .   :  : *. .     .: *      * :  * 

MG03147.1 	FPRTKSREEQIAILQRSSDPKAADKDEGVYDMLVIGGGATGAGVALDAATRGLKVALVER
NCU05454.1	FPQVKSRDQQIADLKKSNSGNKEDE----YDVLVIGAGATGAGVALDAATRGLKVAVVER
FG03249.1 	FSATKARAEALAELRRSGQPNKDTE----YDLLVIGGGATGTGIAIDAITRGLKVALVER
AN1396.1  	FPKIKSRLEQIEDLKRSSSGDDADV----YDLLVIGGGATGSGIALDAATRGLKVAVVER
          	*.  *:* : :  *::*.. .        **:****.****:*:*:** *******:***

MG03147.1 	DDFAAGTSSKSTKLVHGGVRYLEKAVWELDYSQ~YELVKEALKERKYFLQTAPHLSSWLP
NCU05454.1	DDFSSGTSSKSTKLVHGGVRYLEKAVWNLDYAQ~YELVKEALKERKYFLKTAPHLSSWLP
FG03249.1 	DDFSSGTSSKSTKLVHGGVRYLEKAVWNLDYGQ~LQLVMEALRERKTFLNIAPHLSSSLP
AN1396.1  	DDFSAGTSSKSTKLVHGGVRYLEKAVWELDYNQ2YKLVKEALRERKYFLNTAPHLSSWLP
          	***::**********************:*** *  :** ***:*** **: ****** **

MG03147.1 	IMLPLDKWWKVPYYWAGTKFYDFLAGSEGIESSYFLTRSKALDAFPMLKKEGLVGALVYY
NCU05454.1	IMLPLDQWWKVPYYWAGTKFYDFLAGSEGIESSYFLTKSKAIDAFPMLKQTDLVGALVYY
FG03249.1 	ILLPLQKWWEAPYFWAGTKAYDLLAGSQGLESSYYMSKNKAMEAFPKLRKENMVGALVYY
AN1396.1  	IMVPVQKWWQIPYFWAGTKCYDWLAGSEGIESSYFLPKSKAIEAFPMLRKDNVLGAMVYY
          	*::*:::**: **:***** ** ****:*:****::.:.**::*** *:: .::**:***

MG03147.1 	D~GAHNDSRMNVSIAMTAALYGATVVNHMEVTSLTKDANGRLCGATAKDLVPARDGK--P
NCU05454.1	D~GAHNDSRMNVSIGMTAALYGATVVNHMEVTGLQKGENGKLCGATLKDLVTERDGN--E
FG03249.1 	D~GQHNDSRMNVALAMTAAQYGATVLNHVEVTGLEKDANGKINGAQVRDVLASKNGDSAN
AN1396.1  	D1GAHNDSRMNVSLAMTAALYGSTVVNHMEVTGLTKDASGRLNGARLRDCIPERDGQ--G
          	* * ********::.**** **:**:**:***.* *. .*:: **  :* :. ::*.   

MG03147.1 	APEVNIRARCIVNCTGPFSDGIRKLDDSSVQEIVAPAAGVHIVLPGFYSPANMGLIDPQT
NCU05454.1	ATPFNIKAKCIINCTGPFTDSIRKMDDQDCKEIVAPASGVHVILPGYYSPGKMGLIDPST
FG03249.1 	AEPFKVRAKGVVNATGPFTDAIHQMDDPSRKPIVAPASGVHVMLPKDICPNGIGLLDAAT
AN1396.1  	AEEISIRAKGIINATGPFTDSIRKMDDPNTKEIVAPSSGVHVILPGYYSPSNMGLIDPST
          	*  ..::*: ::*.****:*.*:::** . : ****::***::**   .*  :**:*. *

MG03147.1 	SDGRVVFFLPWQGNTIAGTTDDPSTIVKDPIPDEKSIEWILNEIRHYLSPDITVRRGDVL
NCU05454.1	SDGRVIFFLPWQGNTIAGTTDEPATITKNPLPDEKSIQWILNEISHYLSPDINVRRGDVL
FG03249.1 	SDGRVIFVLPWQGFTLAGTTDNPCEVERAPVAQQNDVDFILREVSKLLTPESALSRKDVQ
AN1396.1  	SDGRVIFFLPWQGNTIAGTTDAPTEITRQPQPSEKDIDWILKEIRGYLAPDINVGRSDVL
          	*****:*.***** *:***** *  : : * ..::.:::**.*:   *:*:  : * ** 

MG03147.1 	AAWAG~LRPLVKDPKAKNTEAIVRNHLVDISPSGLVTCAGGKWTTYRQMSEECVDAAIAE
NCU05454.1	AAWSG~LRPLVRDPKAKNTESLVRNHLIDISPSGLITCAGGKWTTYRQMAEECVDAAIKE
FG03249.1 	AAWSG1IRPLVKNPNAKNTESLVRSHLITTSPSGLLTCAGGKWTTYREMAEDTVNEAVKM
AN1396.1  	AAWAG~IRPLVRDPKVKSSEALVRNHLITVSSSGLLTCAGGKWTTYRQMAEEAVDEAIKE
          	***:* :****::*:.*.:*::**.**:  *.***:***********:*:*: *: *:  

MG03147.1 	FKLKPGRVVNAPRVS---GTEEIDDGADLDGSCQTHKVRLIGAHGFSKNLFIHVIQHFGV
NCU05454.1	FNLPVKPIANPPLVS---GTEHVEDDAVLDGSCQTHRVRLIGAHGFSRTLFIHLIQHFGV
FG03249.1 	FELKP-QAVSLPDIS-----GANASGFTTSGLCCTRNIPLIGAHGYSTSLASQLMEMYPI
AN1396.1  	FNLKPCELHDVPDISGVGGRGLVADNASLDGSCQTHQVRLIGAHGWSKTLFINLIQHYGL
          	*:*      . * :*. ..     ..   .* * *:.: ******:* .*  :::: : :

MG03147.1 	ETDVAKHLTESYGDRAWTVAALSKVTDKRFPARGERIHELYPFVDGEVRYAVRHEYAQSA
NCU05454.1	ETEVAKHLTESYGDRAWTVASLCKATNKRFPAKGERIAELYPFVDGEIRYAVRHEYAQTA
FG03249.1 	DADVADHLAHNYGDRAWTVLSTNPSLNTRLVSG-------LPYLEAEVAHGIRNEAACTV
AN1396.1  	ETDVARHLTTSYGDRAWQVAALSSPTQTRFPVRGQRISALYPFIDGEVRFAVRHEYAQTA
          	:::** **: .****** * :     :.*:   ..      *:::.*: ..:*:* * :.

MG03147.1 	IDVVARRTRLAFLNAQATLEVLPKVIDIMTEELKWDKRRQEFEWKET1VKFLESMGLPRP
NCU05454.1	VDVLARRTRLAFLNAQAALEALPKVIDIMADELKWDAKRKDVEWKDT1VAFLESMGLPQP
FG03249.1 	ADIIARRTRLSFLDSSKALQALPRVIDIAATELQWSKARKAQEKADS~IAFLASMGLEQ-
AN1396.1  	VDVIARRTRLAFLNAQAALEALPTVIDLMGEELHWDKNRKEVEWKDS~VQFLASMGLAPD
          	 *::******:**::. :*:.** ***:   **:*.  *:  *  :: : ** ****   

MG03147.1 	LLTVTRKEIEQGKIDLSTSLEYKMYTRHD~KPVEEL----------------~-------
NCU05454.1	MLTTTRQQVEKGKLDWSNSLEWKMYSRHD~KPVDEKERNEQAEIAGRAGTHR1SKGVFVV
FG03249.1 	---MTDAPVQASQQERMPSRQIEHSQQRP~TRVGGLEVDLNGLGSTSSYSKS~RDD----
AN1396.1  	LLNVTRDQVENGKVQQLLASERGAFTRTD1RDRLAAVYCGLSLGSKGLASFW~TRSSFSY
          	  . *   :: .: :   : :     :              .  :    :     .    

MG03147.1 	-----------------------------------
NCU05454.1	LDWTGLDWTGLAGWVGMNVRGSLTLTLFSVGDVVS
FG03249.1 	-----------------------------------
AN1396.1  	A----------------------------------
          	
```
